# Clinical Cases

**Published:** 1887-02

**Authors:** Wm. Jefferson Guernsey

**Affiliations:** Philadelphia


### CLINICAL CASES.

WM. JEFFERSON GUERNSEY, M. D., PHILADELPHIA.

Some time since one of the editors of THE HOMŒOPATHIC PHYSICIAN complained that there was a lack of reports of clinical matter, and stated that ordinary cases would be acceptable. I will herewith present a few of just that sort, and hope others will follow suit.

I. A lady whom I found convalescent from a trouble which had been pronounced ovarian abscess by one of our homœopathic specialists, and who had for months been treated in the usual mongrel or "regular" fashion, and whom I had relieved of the trouble promptly by a discontinuance of the folly and a few careful prescriptions, said that she felt perfectly well, excepting a slight fatigue occasioned by a loss of sleep caused by a fretful child. It is of this child that I have now to speak. It was playful and apparently well during the entire day, sleeping and eating nicely, and presenting absolutely no symptoms to indicate any drug, save in the restless nights. *It would twist and turn and fret from bed-time till morning, and the next day be as lively as ever.* Jalap and Psor. both have this symptom, and I gave the little squirmer *one* powder of Psor. DCM (500,000) Fincke, dry on the tongue. That night the little girl and the mother both rested well all night, and she did not complain of a recurrence of the trouble for several months, until, in fact, she had an attack of croup.

II. G. M., male, æt. seven years. Marked dyspnœa, and every respiration accompanied by a dry cough. Restlessness. Thirst. Delirium at times. *Severe pains in limbs.* Every time he takes a nap (which is always short), *he awakens much worse.* ℞ 4 Lach. CM. (Fincke). The next day improvement was so marked as to permit me to discharge myself. The day following I heard through a member of the family that he was "about well." Let me here remark that aside from the aggravation after sleep, I think the pains in the limbs a good indication for Lachesis. They were severe, and all through the thighs and legs. Whenever I find a "throat case" having these pains, I say confidently to myself, either the left side of the throat is the worse, or the trouble has commenced there, and have *never failed to find it so.* Further than this, I have numbers of times been called to see a person suffering from and complaining mainly of those pains, with fever, which had been preceded by a chill, and slight huskiness or "thickness" of the voice, who would not complain at all of the throat, and perhaps even assert that there was little or no trouble there. Looking, I would find marked ulceration or deposit, and always worse on the left side.

III. W. P., male, æt. forty years, had had neuralgia of face for a month, and had not slept *at all* for the last week. The pain would invariably *compel him to rise and walk about.*

℞. 8 Mag. carb.$^{50m}$ (Swan), beginning about two P. M. He retired that night at nine o'clock and did not waken till morning.

IV. J. W. called last week for medicine for *hæmorrhoids, accompanied by a severe pain in back,* and stated that he wanted the same medicine I had given him for that trouble four years before, as that had relieved the pain in the back *before he left my office.* He had received Æscul.$^{45m}$ (Fincke).

V. E. H., female, eleven years. Throat completely filled with diphtheritic membrane, nose obstructed and having an acrid discharge. Hæmorrhages from nose and throat. The case had been neglected to this sad state of things under the delusion that the girl "had the mumps, and would get well herself." The parents had not looked at her throat and only became alarmed at the offensive odor. One symptom, which I have not yet recorded, decided my prescription, and that was the very *marked torticollis.* Her head was not only drawn out of position, but the deformity was so severe that it could not be overlooked. I gave her Lachnanthes$^{50m}$ (Swan), five powders, and that was all she needed. In a few hours her head was much straighter, and her throat was markedly better the next day. I discharged her well in three days.

